# Determinants of pediatrics emergency mortality at comprehensive specialized hospitals of South nation nationalities and people region, Ethiopia, 2022: unmatched case-control study

**DOI:** 10.1186/s12887-023-04011-3

**Published:** 2023-04-21

**Authors:** Hiwot Tsegaye, Alebachew Demelash, Dawit Aklilu, Bekahegn Girma

**Affiliations:** 1 Dilla University General Hospital, Dilla, Ethiopia; 2 Department of Pediatrics and Child Health Nursing, School of Midwifery and Nursing, College of Medicine and Health Science, Debre Berhan University, Debre Berhan, Ethiopia; 3 Department of Nursing, College of Medicine and Health Science, Dilla University, Dilla, Ethiopia

**Keywords:** Pediatrics mortality, Determinants, Specialized hospitals, Ethiopia

## Abstract

**Background:**

Globally, child mortality is remaining high, especially in sub-Saharan African countries like Ethiopia. Mortality which happens within 24 hours of admission is preventable. However, in Ethiopia little is known regarding pediatric emergency mortality. Therefore, this study was aimed to identify determinants of pediatric emergency mortality at compressive specialized hospitals found in South Nation Nationalities and people region, Southern Ethiopia.

**Methods:**

A facility-based unmatched case-control study was conducted on 344 children (115 cases and 229 controls) at comprehensive specialized hospitals of South Nation Nationalities and people region, Ethiopia. The data collection checklist was checked for its consistency. Data were entered and cleaned for missed values by using Epi Data3.1, then exported to Stata version 14.1 for analysis. Logistic regression was done to identify the significant determinants for pediatric emergency mortality. Finally, AORs at 95% CI and *P*-value < 0.05 were used to declare statistical significance.

**Result:**

A total of 344 charts were reviewed, of which 333 (97%) (112 cases and 221 controls) charts fulfilled the inclusion criteria.. In multivariable analysis, delayed diagnosis and treatment [AOR = 2.088, 95% of CI (1.128, 3.864)], acute respiratory distress syndrome [AOR = 2.804, 95% of CI (1.487, 5.250)], dehydration [AOR = 3.323, 95% of CI (1.260, 8.761)], meningitis [AOR = 5.282, 95% of CI (2.707, 10.310)], sepsis [AOR = 4.224, 95% of CI (2.220, 8.040)], accidental injury [AOR = 3.603, 95% of CI (1.877, 6.916)] and duration of sign/symptoms [AOR = 5.481, 95% of CI (2.457, 12.230)] were significantly associated with pediatric emergency mortality.

**Conclusion:**

In the current study, delayed diagnosis and treatment, acute respiratory distress syndrome, dehydration, sepsis, meningitis, accidental injury and duration of signs/symptoms were significantly associated with pediatric emergency mortality. Healthcare professionals should identify and treat patients early at an emergency department and provide attention to patients with the above diseases. Furthermore, quality care should be provided.

## Introduction

The child mortality rate remains high globally with around 3.1 million neonates, 2.3 million infants, and 2.3 million childhood deaths occurring every year. The mortality rate in children younger than 5 years has dropped [1]. However, the distribution of death in children fewer than 5 years of age is still high; 50% in Sub-Saharan Africa [2]. In Africa, the childhood mortality rate is 15 times more than well-resourced countries [3].

In low-income countries, most of the mortality which happens in the first 24 hours of admission is preventable [4]. Sub-Saharan African facilities have higher patient load and mortality than other regions particularly for pediatric emergency (PE) patients [5].

Out of 60 countries characterized as having high childhood mortality, Ethiopia is among the eight [6, 7]. If situations continue like this, more than 3,084,000 children will die by 2030. Child mortality rate range from as low as 39 per 1000 live births in Addis Ababa to as high as 125 per 1000 live births in Afar [8]. In Ethiopia, over the five-year study period, 4.1% of deaths were recorded at pediatric emergency department. Approximately 32% of the deaths were occurred within ≤24 hours of arrival in PED [9].

Pediatric emergency mortality is one of the most challenging problems for clinicians and a very painful issue in the community. In Ethiopia, Emergency triage assessment and treatment (ETAT) was initiated to decrease the pediatric emergency mortality. However, the mortality is still high [10].

In our country, there are several published articles which conducted to assess the magnitude and factors of under-five and neonatal mortality. However, there is a scarcity of data on determinants of pediatric mortality, especially within 24 hours of admission. Therefore, this study was aimed to identify the determinants of pediatric emergency mortality in the pediatric emergency unit.

## Method

### Study area and period

This study was conducted from Jun 20, 2022 - July 24, 2022 in all comprehensive specialized hospitals of Sothern Nation’s nationalities and peoples region (SNNPR), Ethiopia. SNNPR is one of the administrative regional states in Ethiopia. It is the third largest region in an area out of 11 administrative regions in Ethiopia. It is also the most diverse region in the country in terms of culture, language and ethnicity. There are three comperhensive specialised hospitals in the region. All comperhensive hospitals (Wolaita Sodo University comperhensive specialised hospital (WSUCSH), Wachamo university Nigist Eleni Mohammad memorial comprehensive specialized hospital (WUNEMMCSH) and worabe comprehensive specialized hospital (WUCSH) were included in this study.

WSUCSH is located in Sodo town of Wolaita Zone, which is 380 km away from the national capital. The hospital is serving more than 2 million people in the catchment area in since 1928. The T#town has 28,499 under- five age children and 4576 infants less than 1 year of age [11]. WUNEMMCSH is located in the Hadiya Zone which is 232 kms far from Addis Ababa. Worabe comprehensive specialized hospital is found in the Silte Zone. Worabe town is located 172 kms away from Addis Ababa. It has an estimated total population of 29,600, of which 4618 are under-five children [12].

### Study design

An institution-based unmatched case-control study design was employed to identify determinants of pediatric emergency mortality in all comprehensive specialized hospitals of SNNPR, Ethiopia.

### Source population

All children admitted to pediatric emergency units of all comprehensive specialized hospitals of SNNPR, Ethiopia.

### Study population

All children who died within 24 hr. of admission to the emergency department for the case groups.

All children who survived 24 hrs emergency unit admission in all comprehensive specialized hospitals of the south region for the control group.

### Inclusion and exclusion criteria

#### Inclusion

This study included children aged 29 days to 14 years who died and were discharged within 24 hrs of emergency unit admission.

#### Exclusion

Children with self-discharge (against clinical the decision), children died at arrival and charts of children who died in the intensive care unit (ICU) and pediatric ward were excluded.

### Sample size determination

The sample size was calculated by Epi-Info version 7.2.5 statistical software designed for an unmatched case-control studies. The following assumptions were considered during sample size calculation a 95% level of confidence, a power of 80%, and a case to control ratio of one to two (1:2) with a 2.1 odds ratio. By taking the duration of signs and symptoms ≥2 days as an independent exposure variable which gives the largest sample size [9]. The final sample size after adding a 10% non-response rate was 344 (115 cases and 229 controls).

### Sampling technique and sampling procedure

A systematic random sampling technique was used to select children’s card. The total number of pediatric mortality and discharge at ETAT during the data collection period was estimated from the registration book. Total pediatric mortality within 24 hrs (Cases) in Wachamo, Wolaita Sodo and Worabe respectively were 147, 125 and 73 with a total of 345 children in 2 years. The number of children included in the study from each hospital was calculated using the proportionate to size (PS) allocation technique as follows; ni = (n/N) Ni, where ni = sample size of each hospital, n: total sample size, Ni: population of each hospital and N: total population of three hospitals.

Therefore, based on the above formula sample size for cases from Wachemo, Wolaita Sodo and Worabe hospitals were 49, 42 and 24, respectively. Totally it gives 115 cases. Cases. To get the individual sample units (patient card who died within 24 hours), systematic random sampling technique was used with $\textrm{K}=\left(\frac{N}{n}\right)$ which is $\left(\frac{345}{115}\right)=3.$

Hence, about 115 children who died within 24 hours were selected using systematic random sampling technique every 3 chart interval after the first chart was determined by lottery method which was the second children who died within 24 hours in registration book.

**Fig. 1:**
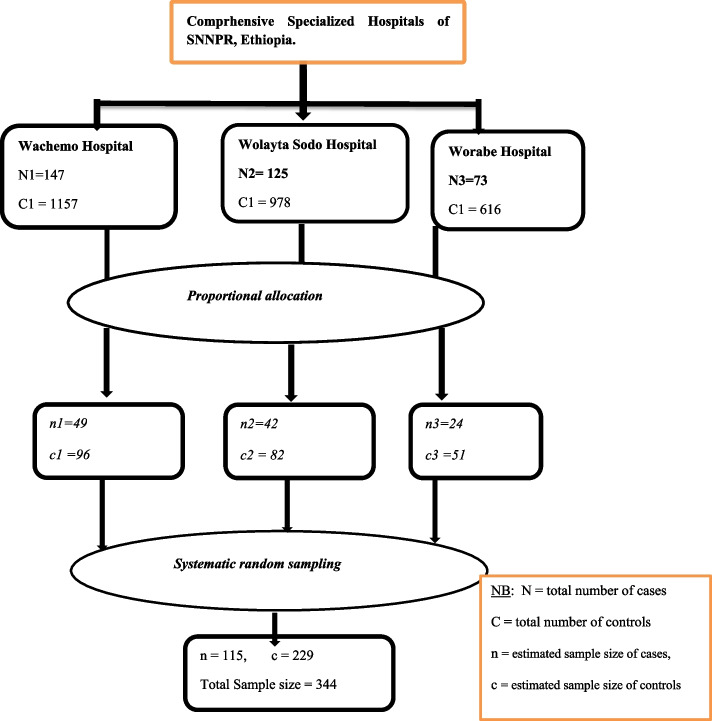
Sampling procedure for pediatric emergency mortality, 2022

The total number of patients discharged from ETAT within 24 hours (controls) in Wachamo, Wolaita Sodo and Worabe in the past 2 years was 1157, 978 and 616 respectively. Subsequently, based on the above formula the sample size for controls from Wachemo, Wolaita Sodo and Worabe hospitals was 96, 82 and 51. It gives the total number of 229 controls every 12 children (Fig. 1).

### Operational definition

Delay in seeking a health facility is as staying home for 48 hours or more before consulting any formal healthcare facility [13].

An incomplete chart means a chart without triage paper and death summary.

Poor nursing follow-up means not giving medication timely and also not taking vital signs within appropriate time interval.

Pediatrics emergency mortality is a record of death that occurred children aged 29 days − 14 years with in 24 hrs of admission to the emergency unit [14].

Treatment delay is not starting any treatment within 1 hour of emergency unit admission [15].

### Variables

#### Dependent variable

- ❖ Pediatrics death in an emergency unit (≤24 hours.)

#### Independent variable

- ➢ Socio-demographic and environmental characteristics: age, sex, birth weight, area, season
- ➢ Disease factors: medical emergency disease and surgical cases, accidental/injuries, sign and symptoms, disease severity
- ➢ Delay in seeking health facility: referral source, duration of signs and symptoms, treatment delay
- ➢ Previous hospital history: repeated hospital visit, previous hospital admission, congenital problem
- ➢ Poor nursing follow-up-Complete vital signs and medication chart

### Data collection tools

The data were collected by 9 BSC nurses and 3 supervisors using a structured checklist from pediatric patient’s chart. The checklist was adapted from different literatures [9, 16, 17]. The data collection checklist includes socio-demographic characteristics, clinical presenting features, and the main medical cause of mortality. The disease cause of mortality was defined according to the HMIS and IDC at the hospital level across the country with related different pediatric age groups.

### Data quality assurance

Two-day training was given by the principal investigator on the data extraction checklist and procedure. Before actual data collection, the checklist was pre-tested at WSCSH to check the appropriateness of the checklist with 5% of the sample size. After the pretest, the tool was checked for its consistency by the principal investigator. Finally, the coded data were entered into password-protected computer statistical software.

### Data processing and analysis

Data were entered and cleaned for missed values by using EpiData3.1 then exported to Stata version 14.1 for analysis. The description of means, simple frequencies, proportions, and SD of the given data on each variable were computed to compare the exposure status between cases and controls. The normality of the data was tested by skewness and kurtosis test. The degree of association between each independent and dependent variable was assessed by the logistic regression analysis model. The variables with *p*-value < 0.25 during bivariate analysis were selected as candidate variables for multivariable analysis. Model goodness of fit was tested by Hosmer-Lemeshow’s goodness of fit test with the estat gof command on Stata. Multi-Collinearity was checked by the variance inflation factor (VIF) test. An adjusted odds ratio (AORs) at 95% CI was used to show the strength of the association, and statistical significance was declared at a *p*-value of < 0.05 as a determinant for pediatric mortality.

## Result

**Fig. 2:**
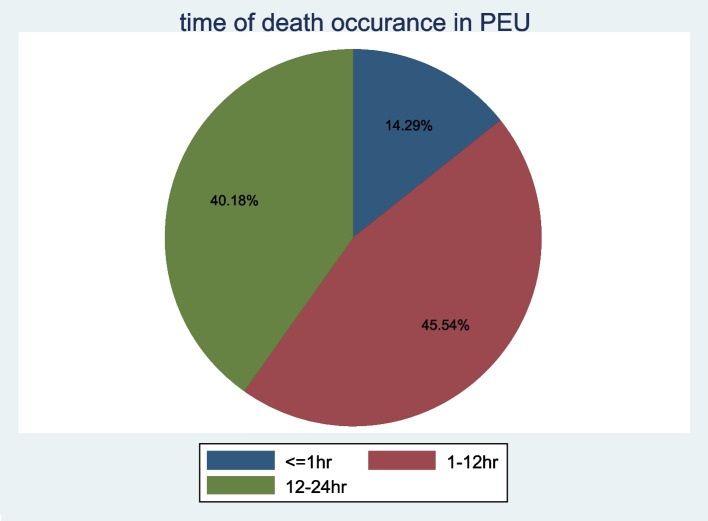
Time interval of death documented in PED at all comprehensive specialized hospitals in SNNPR, Ethiopia, 2022

A total of 344 charts were reviewed, of these 333 (97%) (112 cases and 221 controls) charts fulfilled the criteria. More deaths occurred in males (53.6%), with a male to female ratio of 1.2:1. Approximately 45.5% of deaths were documented as early death (within 1–12 h of arrival in the pediatric emergency department) (Table 1) (Fig. 2).

**Table 1:** Socio-demographic characteristics of children admitted in the emergency unit at all comprehensive specialized hospitals in SNNPR, Ethiopia, 2022

Variables	Category	The Outcome of pediatrics	
		Case = 112 (%)	Controls = 221 (%)
Age	Infant	59 (52.7)	89 (40.3)
	Preschool	31 (27.7)	67 (30.3)
	School age	22 (19.6)	65 (29.4)
Sex	Female	52 (46.4)	125 (56.6)
	Male	60 (53.6)	96 (48.3)
WFA	Appropriate	57 (55.9)	99 (44.8)
	Under weight	45 (44.1)	122 (55.2)
Residence	Urban	64 (57.1)	132 (59.7)
	Rural	48 (42.9)	89 (40.3)
Season	Summer	59 (52.7)	106 (48)
	Winter	53 (47.3)	115 (52)

The mean ages of cases were 70 (S.D ± 9) months and 49 (S.D. ± 7) months for controls. Also, the mean weight of children in cases and controls was 11.21 (S.D ± 3.47) and 12.73 (S.D ± 3.71) kilogram respectively. Of those cases 55.9% had appropriate weight for age but 50.7% of controls were underweight.

One hundred one (90.2%) of the cases and one hundred nine (49.3%) of the controls visited health facility after one day of signs and/or symptoms. More than half of cases came from urban region with 40.2% referrals from internal health institutions and 59.7% of controls from an urban with 38.9% self-referral. Concerning treatment delay, 64 (57.1%) of cases and 160 (72.4%) of controls were taken medication timely (Table 1).

**Fig. 3:**
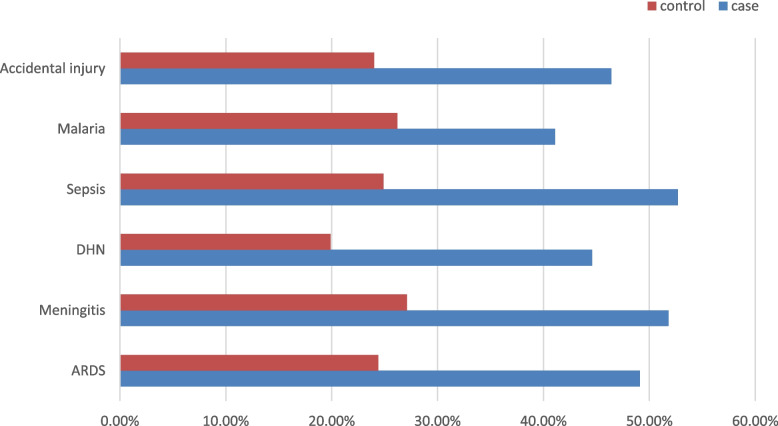
Disease occurance of case and controls as determinant of pediatrics emergency unit mortality, 2022

Eighty-five (75.9%) of cases presented with more than two sign/symptoms and for controls, 95 (43%) presented with more than two signs/symptoms (Table 2). Surgical cases including intestinal perforation and obstruction contributed to 5.4% of cases and 5.4% of control plus to these accidental cases including motor accidents and poisoning were presented in 34.8% of cases and 24% of controls. The contribution of secondary causes for death from both DHN and shock was 77.7%. Comorbidity contributed 49.1% of death (Fig. 3)

**Table 2:** Frequency distribution of presenting sign and symptoms in PED at all comprehensive specialized hospitals, SNNPR, Ethiopia, 2022

Variables	The Outcome of pediatrics		
	Category	Case = 112 (%)	Controls = 221 (%)
Fever	No	57 (50.9)	121 (54.8)
	Yes	55 (49.1)	100 (45.2)
Diarrhea	No	42 (37.0)	131 (59.3)
	Yes	70 (62.0)	90 (40.7)
Cough	No	60 (53.6)	153 (69.2)
	Yes	52 (57.4)	68 (30.8)
Shortness of breathing	No	81 (72.3)	164 (74.2)
	Yes	32 (27.7)	57 (25.8)
Fast breathing	No	45 (40.2)	136 (61.5)
	Yes	67 (59.8)	85 (38.5)
Vomiting	No	67 (59.8)	144 (65.2)
	Yes	45 (40.2)	77 (34.8)
Abnormal body movement	No	60 (53.6)	123 (55.7)
	Yes	52 (46.4)	98 (44.3)
Other	No	96 (85.7)	184 (83.3)
	Yes	16 (14.3)	37 (16.7)

While examining specific charts for a specific disease, from cases 62 (55.4%) used appropriate charts regarding with disease and 179 (81%) was appropriate for controls. When vital sign charts completeness evaluated 68 (60.7%) cases and 154 (69.7%) controls completed. Seventy-six (67.9%) of the cases and 151 (68.3%) of controls, were have completed medication chart.

### Determinants associated with pediatric emergency mortality

After bivariate analysis 13 variables were selected for multivariable analysis. However, only the seven remained significant determinates which were acute respiratory distress syndrome (ARDS), dehydration (DHN), meningitis, sepsis, unintentional accident, duration of sign/symptoms (> = 1 day) and the right time of medication (treatment delay).

In this study, patients with treatment delay were 2 times more likely to die than without delay (AOR = 2.088, CI = 1.128–3.864). Pediatrics who had ARDS were 2.8 times more likely to die in the emergency unit than pediatrics who did not diagnose with ARDS (AOR =2.804, CI = 1.497–5.250). Children diagnosed with meningitis had 5.2 times more risk for mortality (AOR = 5.282, CI = 2.707–10.310), and those pediatrics presented with sepsis had 4.8 times more odds of death within 24 hours of hospital visit as compared to their counterparts (AOR = 4.224, CI = 2.220–8.040).

Children who visit the pediatric emergency unit with unintentional accidental injury had 3.6 times more risk for mortality (AOR = 3.603, CI = 1.877–6.916) in comparison to their opposite group. Patients whose duration of signs/symptoms was greater than 24 hours before presenting to the emergency unit had a 5.8 times more risk for mortality in the pediatric emergency unit (AOR = 5.481, CI = 2.457–12.230) as compared to their counterparts (Table 3).

**Table 3:** Factors (crude and adjusted odds ratios and confidence intervals) associated with pediatric emergency unit mortality at all compressive specialized hospitals in SNNPR, Ethiopia, 2022

Variables		Death				
		Yes	No	COR (CI, 95%)	AOR (CI, 95%)	***P***-value
Sex	F	125	96	1.502 (0.952, 2.372)	1.611 (0.865, 2.998)	0.133
	M	52	60			
Fast breathing	Yes	60	52	1.846 (1.166, 2.924)	1.718(.903,3.267)	0.099
	No	85	136			
Malaria	Yes	43	69	1.751 (1.079, 2.843)	1.725(.878,3.387)	0.114
	No	58	163			
**DHN**	Yes	55	57	**3.882 (2.363,6.376)**	**4.883 (2.493,9.563)**	**0.00**
	No	44	177			
**Meningitis**	Yes	59	53	**2.987 (1.858, 4.803)**	**5.282 (2.707,10.310)**	**0.000**
	No	60	161			
Pneumonia	Yes	80	32	2.917 (1.791,4.751)	1.345(.694,2.606)	0.379
	No	102	119			
**Sepsis**	Yes	59	53	**3.360 (2.079, 5.431)**	**4.224 (2.220,8.040)**	**0.00**
	No	55	166			
**ARDS**	Yes	47	65	**2.236 (1.377,3.631)**	**2.804 (1.497,5.250)**	**0.001**
	No	54	167			
**Accidental**	Yes	52	60	**2.747 (1.695, 4.453)**	**3.603 (1.877,6.916)**	**0.00**
	No	53	168			
Malnutrition	Yes	50	62	1.704 (1.086,3.248)	1.604(.856,3.004)	0.14
	No	71	150			
Previous admission	Yes	45	67	1.932 (1.192, 3.133)	1.837(.935,3.607)	0.077
	No	57	164			
**Duration of sign/ symptoms**	> 1 day	31	81	**0.283 (.173,.463)**	**5.481 (2.457,12.230)**	**0.00**
	<=1 day	127	98			
**Treatment delay**	Yes	61	51	**0.953 (.603,1.505)**	**2.088 (1.128,3.864)**	**0.019**
	No	123	98			

## Discussion

In this study, seven determinants ARDS, DHN, meningitis, sepsis, unintentional accident, duration of sign/symptoms (> = 1 day), and treatment delay were identified for pediatric emergency unit mortality.

Patients who were not early identified and treated had 2 times more risk to die than those without delay. This finding is consistent with a study done in central America and Tanzania [18, 19]. This might be due to many hospitals in low-income countries serve large number of patients and has few staffs, so patients often have to wait before being assessed and treated which leads to deterioration of patient condition [19]. Furthermore, in most the African countries including Ethiopia, children are not checked before a senior health worker examines them; as a result, some seriously ill patients have to wait a very long time before they are seen and treated [20].

In the present study, children with ARDS had 2.8 times more risk for mortality at the emergency unit than pediatrics who were not diagnosed with ARDS. This is supported by studies conducted in South Asia, Sub-Saharan Africa, and Tikur Anbessa Hospital, Ethiopia [9, 21, 22]. This similarity might be due to ARDS can cause multiple organ failure, including lung collapse which is responsible for most of the child early mortality [23].

In the current study, children diagnosed with meningitis and sepsis had 5.2 and 4.8 times more risk for mortality as compared to their counterparts, respectively. This finding is supported by studies done in China, India, Ghana and World Health Organization (WHO) reports [24–27]. This similarity might be due to both meningitis and sepsis being more common in low and middle-income countries (85%) and they have a potential for organ failure and they need early diagnosis and treatment but as mentioned above in low and middle-income countries including Ethiopia there is a high rate of treatment delay for children. Moreover, in most countries, bacterial antibiotic resistance is increased which leads the child into deteriorating clinical condition, and finally to death.

Children with dehydration had 4.8 times more odds of death as compared to their comparison group. This finding is consistent with studies conducted in Uganda and Brazil [28, 29], This similarity might be due to there is lack of oral rehydration therapy (ORT) and low immunization coverage in developing countries. Also, it might be due to dehydration can cause severe metabolic and electrolyte disturbance and treatment delay for dehydration in Ethiopia is high which leads to mortality [30].

Children who visit the pediatric emergency unit with unintentional accidental injury had 3.6 times more risk for mortality. This finding is consistent with previous studies conducted in Bahir Dar, Addis Ababa, and low- and middle-income settings [31–33]. This might be because of accidental injury being able to cause excessive blood loss and there is the low quality of first aid service at the time of an accident in Africa. Moreover, might be due to in sub-Saharan settings accidental injury is among the leading causes of mortality for children [33].

Lastly, children whose signs/symptoms greater than 24 hours before presenting to the emergency unit had 5.8 times more odds for mortality. This finding is consistent with studies done in Addis Ababa and Nepal [9, 34]. This could be due to timely and appropriate treatment within 24 hours of the onset of illness symptoms can reduce severe morbidity and mortality among children in most African countries. Moreover, in Africa most families visit health institutions at the end after religious prayer and traditional medicine treatment which makes unable to early identify critically ill children and poor treatment seeking behavior of caregivers [35].

In this study, incomplete chart record and the presence of multiple diagnoses were the limitations.

## Conclusion

The majority of pediatric emergency mortality occurs within 24 hours of admission. In this study, ARDS, sepsis, meningitis and dehydration, delayed diagnosis and treatment, duration of signs and symptoms, and accidental injury were also significantly associated with early pediatric emergency mortality. To decrease this mortality, ETAT training should be provided for healthcare providers and its implementation should be strengthened. Furthermore, healthcare professionals should provide special attention and provide early treatment for children with ARDS, sepsis, meningitis and dehydration. Lastly, the concerned bodies also design strategies to increase the community health-seeking behavior.

## Data Availability

The data used in this manuscript are available upon reasonable request by contacting the corresponding authors via email.

## Abbreviations

AHR: Adjusted Hazard Ratio
ARDS: Acute respiratory Distress Syndrome
CHR: Crude Hazard Ratio
DHN: Dehydration
NICU: Neonatal Intensive Care Unit
ETAT: Emergency triage assessment and treatment
PED: Pediatric emergency department
SNNP: South nation and nationalities people
WSUCSH: Wolayita Sodo University Comprehensive Specialized Hospital
WUNEMMCSH: Wachemo University Nigist Eleni Mohammed Memorial comprehensive specialized hospital
